# Genetic Modification of Hematopoietic Stem Cells as a Therapy for HIV/AIDS

**DOI:** 10.3390/v5122946

**Published:** 2013-11-28

**Authors:** Patrick Younan, John Kowalski, Hans-Peter Kiem

**Affiliations:** 1 Clinical Research Division, Fred Hutchinson Cancer Research Center, Seattle, WA 98109, USA; E-Mails: pyounan@fhcrc.org; jkowalsk@fhcrc.org; hkiem@fhcrc.org; 2 Department of Medicine, University of Washington, Seattle, WA 98195, USA

**Keywords:** HIV/AIDS, Gene Therapy, Stem Cell Transplantation

## Abstract

The combination of genetic modification and hematopoietic stem cell (HSC) transplantation may provide the necessary means to develop an alternative treatment option to conventional antiretroviral therapy. As HSCs give rise to all hematopoietic cell types susceptible to HIV infection, modification of HSCs is an ideal strategy for the development of infection-resistant immune cell populations. Although promising results have been obtained in multiple animal models, additional evidence is needed to convincingly demonstrate the feasibility of this approach as a treatment of HIV-1 infected patients. Here, we review the potential of HSC transplantation and the recently identified limitations of this approach. Using the Berlin Patient as a model for a functional cure, we contrast the confines of autologous versus allogeneic transplantation. Finally, we suggest that although autologous, gene-modified HSC-transplantation may significantly reduce plasma viremia, reaching the lower detection limits currently obtainable through daily HAART will remain a challenging endeavor that will require innovative combinatorial therapies.

## 1. Introduction

Highly-Active-Anti-Retroviral Therapy (HAART) is debatably one of the greatest breakthroughs of modern medicine over the last three decades [1]. Where HIV-infection was once associated with a significant decline in the quality of life and impending mortality within a 10 year span, HIV infection is now considered a highly treatable disease when antiretroviral therapy is available [2,3]. Although this achievement cannot be understated, it has long been known that HAART does not equate a cure [4,5,6], and hence, a continued effort to develop innovative new therapies to achieve the ultimate goal of curing HIV-1 infected patients is still needed [7]. 

In 2009, a study by Hutter *et al.* described the initial results from a single HIV-1-infected patient suffering from acute myeloid lymphoma received allogeneic hematopoietic stem cell transplantation from a CCR5‑/‑ donor [8]. This patient, known as the Berlin Patient, was shown to be effectively cured of HIV-infection in subsequent studies with no detectable viral replication being observed in any of the lymphoid tissues four years following transplantation [9,10,11]. These findings have spawned the development of a multitude of approaches aimed at rendering HSC-derived cells resistant to HIV-infection (Reviewed in [12,13]. 

Indeed several methods have been shown to be highly effective at rendering cell lines, primary cells and HSC-derived cells resistant to HIV; however, the utility of genetically modifying would-be target cells as a potential end all cure has yet to be established. Hence, regardless of the method being employed to protect cells from infection (be it expression of restriction factors or disruption of co-receptors with sequence specific nucleases or other method) the efficacy of stem cell transplantation as a curative approach for HIV-1-infected patients by-in-large remains to be determined [12,13].

In this review, we will examine the potential of hematopoietic stem cell transplantation (HSCT) as a possible curative approach for treatment of HIV-1 infected patients. Although limited in vivo studies have been conducted to date, several notable findings have provided encouraging results that have paved the way for multiple phase I clinical trials. We will examine the potential of autologous HSCT and contrast the limitations of this approach versus allogeneic transplantation. Finally, we will examine the ‘X-factor’; the development of an enhanced immunological response against HIV following the transplantation of genetically modified, infection-resistant immune cell populations.

## 2. Preliminary Results

In the pursuit of a “one time only” treatment utilizing HSC-based gene therapy, the current progress suggests intriguing yet inconclusive results as a relatively small number of in vivo studies have been conducted thus far [14]. Currently, multiple groups are pursuing research in murine and nonhuman primate models with the goal of developing novel genetic modification strategies coupled with HSC transplantation (HSCT) methods as an alternative to conventional daily antiretroviral therapy. In addition, previous and ongoing human clinical trials have paved the way for future gene therapy clinical trials for the treatment of HIV-infected individuals [14]. Although the efficacy of this approach has yet to be shown to be a viable option, the possibility of continued development of this therapeutic approach may in the future lead to a functional cure of HIV-1-infected patients [14,15,16,17]. In this section, we will examine recent studies conducted in murine and nonhuman primate animal models and compare and contrast recent findings with regards to the utility of allogeneic and autologous HSCT as a potential curative therapy for HIV-1-infected patients.

### 2.1. Studies in Murine and Nonhuman Primate Models

Multiple approaches have been developed to inhibit HIV at various stages of the viral life-cycle with some studies combining multiple barriers to prevent viral replication. Studies conducted in murine models have demonstrated that following HIV-challenge of humanized mice, CCR5 disruption led to a significant increase in the percentage of genetically modified CD4+ T-cells demonstrating a clear selective advantage over non-modified cells [18]. Similarly, infection of humanized mice transplanted with CD4+ T-cells expressing the mC46 fusion inhibitor peptide resulted in the positive selection of genetically modified CD4+ T-cells following HIV-challenge [19]. Studies by Walker et al., in which human CD34+ cells were genetically modified via a retrovirus encoding several therapeutic elements; including a TAR decoy, TRIM5α isoform, and a short hairpin RNA (shRNA) targeting CCR5; demonstrated similar results [20]. 

Concerning, however, was the finding that plasma viremia remained relatively high (albeit significantly reduced) despite the significant increase in infection resistant CD4+ T-cells in all studies [20]. This contrasts ex vivo studies in which, purified genetically resistant immune cells exhibit a multi-log reduction in viral titers [20]. Although the source of the residual reservoir was not examined in these murine studies, our own studies in the SHIV-pigtailed macaque model corroborate these findings that suggest although there is a selective advantage for genetically modified cells following infection, viral reservoirs are able to establish despite the infusion of protected cells prior to HIV/SHIV challenge [21]. 

Unlike findings in murine models, we showed that following the acute phase of infection, non-modified cells make a steady, yet remarkable recovery in the pigtailed macaque model [21]. These findings suggest that even with partial engraftment of infection-resistant immune cells, a positive bystander effect may be observed. The potential implications of the increase in non-modified CD4+ T-cells, however, would indicate that a source for viral reservoir replenishment will be maintained. However, in our NHP studies, we did detect a significant decrease in plasma viremia of between 300–1400-fold with the greater reduction occurring in the macaque with the highest levels of genetically modified cells [21]. As cited in several in vivo studies, the percentage of genetically modified cells that is needed to completely suppress viral replication will likely play an important role in determining the clinical feasibility of this approach. The incorporation of an in vivo selection cassette, in which chemotherapeutic agents are used to positively select genetically modified cells, has previously been shown to be a highly effective means for increasing the percentage of modified cells [16,21].

### 2.2. Hematopoietic Stem cell Transplantation in HIV-1 Infected Patients & Ongoing Clinical Trials

HIV-1-infected patients suffering from any of a number of types of cancer (most commonly leukemia and lymphoma) have been undergoing allogeneic transplantation for well over three decades [14,22,23]. Until 2009, and the first report of the Berlin Patient [8], the utility of HSC transplantation as a curative approach for HIV-1 infected patients was unknown. Numerous procedures were performed in the 2000s, with the addition of potent anti-retroviral drugs. Unfortunately, any patients exhibiting a drop in HIV-DNA copies (as expected post-myeloablation of infected cell populations) either succumbed to disease soon thereafter, or experienced a rebound of HIV after HAART was stopped [14,24,25]. For an in depth history of results and analysis for these early allogeneic transplants we direct the reader to reference [14]. In 2009, the first report of an HSCT transplantation leading to the cure of an HIV-1-infected patient, the Berlin Patient, demonstrated the utility of HSCT transplantation as a curative approach for HIV-1-infected patients [8]. It was these findings that have ignited hope of an impending therapy that would effectively eliminate the need for a lifetime of HAART.

Two HIV-1-infected patients in 2010, here known as the Boston patients, heterozygous for the CCR5 allele that had undergone allogeneic transplantation from a wild-type (homozygous for full length CCR5) donor following reduced intensity conditioning maintained undetectable levels of proviral DNA or RNA in the peripheral blood [26]. These patients, who were kept on HAART throughout the transplantation procedure, have been virus-free for nearly 4 years following HSCT. Although HAART has only recently ceased and data pertaining to this cessation has not been released, the initial results thus far suggest that both patients remain virus free in the peripheral reservoir several months following their treatment interruption [26]. 

This underlines a primary question with ongoing clinical trials; in order for the efficacy of the treatment to be quantified, patients must cease HAART [14,26,27]. In both the Boston and Berlin patient studies, clearance of detectable virus coincided with full donor chimerism. In addition to full donor chimerism, incidences of GVHD occurred in both Boston patients. It is this GVHD which is postulated to have played an anti-lymphopoietic role in peripheral blood reservoir depletion of virus through elimination of infected cells harboring proviral DNA [26]. The results of the Boston study, in which patients were transplanted with wild-type allogeneic HSCs, suggests that with completely suppressive antiretroviral therapy, genetic modification of transplanted HSCs (in an allogeneic setting) may not be necessary. For this therapeutic approach to succeed, it will be imperative to limit viral replication during transplantation and in the subsequent months in which donor cells replace all host cells including those harboring latent reservoirs. Hence, the timing of antiretroviral treatment interruption will be critical to the successful outcome following transplantation of non-modified, wild-type HSCs. This approach, however, may be ineffective as previously conducted allo-transplant studies have demonstrated that patients who appear to exhibit complete suppression of HIV on HAART exhibited a rebound of the virus upon cessation of treatment [28,29].

**Figure 1:**
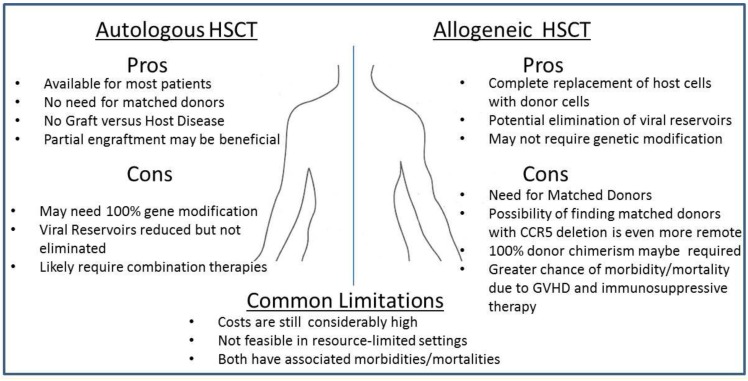
**Pros- and Cons- of Autologous and Allogeneic Transplantation for the treatment of HIV-1 infected patients.** Both autologous and allogeneic transplantation studies are currently moving forward in clinical trials. Here, we list several of the potential positive and negative drawbacks of each transplantation procedure. Evidence for the potential successful outcome following allogeneic transplantation has been observed in one HIV-1 infected patient, known as the Berlin Patient, who was successfully cured of HIV following allogeneic transplantation from a CCR5-/- donor. Limited, yet promising studies have suggested that autologous transplantation of genetically modified HSCs may give rise to infection-resistant immune cell populations that in turn may have the capacity to reduce viral pathogenesis. However, studies in murine and nonhuman primates remain inconclusive as plasma viremia has thus far remained readily detectable in animals challenged following transplantation of genetically modified cells.

In contrast to the results from the aforementioned allogeneic transplants, following autologous transplantation, viral DNA and/or RNA remains detectable by sensitive methods [27]. These findings lead to the conclusion that a myeloablative treatment and subsequent autologous transplantation may only transiently reduce viral reservoirs. If this lingering viremia is in fact due to resting CD4+ T cells harboring virus [27], myeloablation via chemotherapy would not eradicate these cells as they lack the metabolic activity that chemo-induced cell toxicity requires. This would suggest that genetic modification of HSCs in an autologous transplant setting would be necessary to achieve therapeutic value without the aforementioned GVHD effect of an allogeneic transplant. Fortunately, it has been observed that even with extremely low levels of genetically modified cells in an autologous transplant setting, some therapeutic value can be achieved [30]. The benefits and limitations of allo- versus autologous transplantation are summarized in Figure 1.

## 3. Establishing an ‘AIDS Patient Model’ in Nonhuman Primates

Although several HSCT clinical trials have previously been conducted or are currently underway, using both modified and non-modified HSCs and in both the autologous and allogeneic setting, there remains a significant gap in our knowledge with regards to what is needed to achieve a functional or sterilizing cure [14]. For the most part, nonhuman primate models offer an excellent *in vivo* model for studying novel viral eradication strategies [31]. NHPs will likely play a critical role in establishing the threshold of modified cells needed to control plasma viremia to levels that parallel those found in HIV-1-infected patients on conventional HAART [21,31]. In addition, the use of NHPs will undoubtedly provide a unique opportunity to assess viral reservoirs that are refractory to myeloablative irradiation. Determining the source of these resistant cells may open the realm of developing new therapeutics to target these resistant reservoirs, which may be more effectively targeted in combination with irradiation. 

### 3.1. Autologous Transplantation and the Missing Ingredient: Graft-Versus-Host Disease?

As indicated previously, autologous HSCT has been used to treat patients suffering from numerous cancers; to date, not one HIV-1-infected patient has effectively been cured of HIV following autologous HSCT. This begs the question: Can genetically modified HSCs provide the necessary protection following engraftment to maintain plasma viremia at or near undetectable levels? In both aforementioned allogeneic clinical trials, mixed chimerism resulted in detectable viral nucleic acid in PBMCs [8,26]. Proviral DNA only disappeared from peripheral blood mononuclear cells (PBMCs) with the onset of full donor chimerism. The complete elimination and replacement of putative provirus harboring cells, be it CD4+ T-cells or macrophages, will not occur in an autologous transplantation setting; hence, unless myeloablative chemotherapy treatment and and/or the addition of other depletion therapies (e.g. anti-thymocyte globulin) completely eliminates all viral reservoirs, autologous transplantation will remain an ineffective therapy against HIV [27]. Indeed, several studies have shown that autologous transplantation of HIV+-individuals does not result in prolonged absence of proviral DNA in peripheral CD4+ T-cells [27]. 

A central concern regarding high-dose chemotherapy and autologous HSCT in HIV-1-infected patients with lymphoma is the combinatorial effects that may lead to further depletion caused by the chemotherapy and pre-existing immune deficiencies [17]. However, minimal variations in the recovery rate of CD4+ T-cell subsets have been reported between HIV+ and HIV--patients with lymphoma [15,17,32,33,34]. Similarly, signal joint T-cell receptor excision circles (sjTRECs) kinetics was nearly equivalent in both patient populations [15,17,35]. Importantly, proviral DNA content in peripheral blood has previously been shown to be significantly reduced following autologous HSCT in HIV-1-infected patients [17]; albeit with continued HAART in the ensuing months following HSCT.

Although limited, studies in HIV-1-infected lymphoma patients have demonstrated the benefits of autologous stem cell transplantation in terms of the potential benefits of the reconstituted immune system. Full CD4+ T-cell recovery has been observed following autologous transplantation in HIV-1-infected patients with lymphoma [15]. However, it currently unknown if the recovery observed was due to redistribution or peripheral expansion of existing CD4+ T-cell populations [15]. Additionally, thymic selection and TCR rearrangement may have contributed to the recovery of the CD4+ T-cell repertoire although this is less likely in adults due to thymic involution suggesting post-thymic T cell regeneration [35]. 

HIV-1-infected patients have been shown to maintain elevated Interleukin (IL)-7 levels, a cytokine with an important role in the homeostatic response to T-cell depletion [36,37,38,38,39,40,38,38]. The increase in IL-7 has been linked with HIV-infection induced CD4+ T-cell depletion and may provide the necessary signals for thymic-independent T cell regeneration [15,36,41,42,43]. Studies have suggested that serum IL-7 levels are critical for the post- recovery of CD4+ T-cells following autologous HSCT in HIV-1-infected patients [15]. In support of the role of IL-7 in HIV-1-infected patients receiving HAART, IL-7 levels drop to baseline following the recovery of CD4+ T-cells independently of the mechanisms involved in the recovery of CD4+ T-cells (e.g. independent of thymic or peripheral expansion) [15,44,45]. Autologous HSCT has proven to be an effective and safe means for treating HIV-1-infected patients with lymphoma as treatment does not contribute to additional detrimental immune impairment and does not promote viral replication or the seeding of new viral reservoirs [17]. As previously indicated, to date there are promising results from in vivo studies, in which genetically modified cells were transplanted in animals (murine and NHPs) prior to HIV-1/SHIV-challenge. In all cases, however, regardless of the percentage of genetically modified cells, plasma viremia remained detectable in all published in vivo studies. When considering transplantation of fully suppressed patients on HAART, the engraftment of genetically modified autologous HSCs that give rise to infection resistant immune cell populations may enable a patient’s own immune system to completely suppress viral replication; in essence, it may be feasible to artificially create a natural controller phenotype.

### 3.2. Reduction of Viral Reservoirs following Myeloablative Conditioning & Non-HSC Derived Macrophages

Latently infected cells, which serve as the ultimate obstacle to an effective cure, are characterized by the maintenance of proviral DNA in a dormant state [6,44,45,46]. A small subset of memory CD4+ T-cells (and potentially other target cells) are the suspected cell types that harbor proviral DNA [44,45]. Following cellular activation, viral replication may occur unimpeded causing the subsequent seeding of new infections; hence, adherence to HAART drugs therapy regimens is paramount to the eventual decay of the viral reservoirs. Based on prediction models, however, the time to eradication for patients on completely suppressive therapies exceeds 30-60 years [47,48,49]. Hence, alternative methods that are capable of significantly reducing latently infected cells will be of critical importance to achieving a curative therapy.

As indicated previously, myeloablation followed by autologous transplantation does not result in the total eradication of latently infected cells despite patients remaining on HAART throughout the transplant procedure [27]. Analysis of PBMCs following transplantation demonstrated that despite continued HAART, proviral DNA can be readily detected in the months following autologous transplantation [28]. Identifying proviral DNA containing cell types that are refractory to chemotherapy and/or irradiation may prove highly beneficial in terms of the development of novel therapies aimed at targeting these remaining infected cells. 

Although it has been generally accepted that tissue macrophages and dendritic cells originate from HSCs [50,51], recent studies have suggested an alternative developmental pathway which features the development of macrophages early during embryogenesis and prior to the development of HSCs [52,53]. These cells, which are genetically distinct from HSC progeny, are thought to contribute a significant portion of tissue macrophages in several tissues including liver Kupffer cells, epidermal Langerhans cells and microglia [54,55,56,57,58]. This may pose a significant obstacle in establishing resistant reservoir following autologous stem cell transplantation. Although genetically modified HSC-derived cells may develop into infection resistant immune cell populations, these non-HSC derived macrophage cells may continue to harbor proviral DNA as they will remain susceptible to infection. While it is conceivable that tissue macrophages may arise from two independent multipotent progenitors, additional research in needed to determine the identity and source of the progenitors that give rise to the non-HSC derived subset of tissue macrophages and to determine the effects of chemotreatment and irradiation in terms complete abolishment or recovery of these ‘HSC-like’ cells. 

### 3.3. The X-factor: Harnessing the Immune System

Our recent studies in pigtailed macaques were the first to demonstrate that in addition to genetically modified cells being resistant to infection in vivo, these cells in turn provide the necessary cellular functions required for an enhanced immune response against the SHIV-challenge virus as determined by the maintenance of SHIV-specific, gene modified CD4+ T-cells and improved CTL and antibody responses [21]. A key question has emerged from these findings with regards to the potential of harnessing the immune response to control viral replication in the absence of HAART. It is conceivable that by providing conditions that favor an improved, infection resistant-immune response against HIV that a natural controller phenotype can be achieved following HSCT. Tipping the balance in host’s favor would require a significant reduction in viral reservoirs in addition to the development of infection resistant immune cell populations. Persistent, low level viral replication will undoubtedly be a significant detriment as this will result in continued immune stimulation, which in turn will cause damaging effects in terms of destruction of lymphoid tissue and the eventual development of immune exhaustion [59,60,61,62].

Prolonged viral suppression with HAART typically reduces HIV-specific immune responses presumably due to the disappearance of viral antigens [63,64]. However, upon treatment interruption, HIV-specific CD4+ T-cells are susceptible to infection, and hence, their critically important helper function is lost. In patients that naturally control viral replication, both HIV-specific CD4+ and CD8+ T-cell responders are readily detectable and maintained throughout infection [63,64]. While there is a gradual attrition of HIV-specific CD4+ T-cells responders in the Gut-Associate-Lymphoid-Tissue (GALT) of HIV-1 infected patients on HAART, these responses are typically robust in spontaneous controllers [65]. 

**Figure 2:**
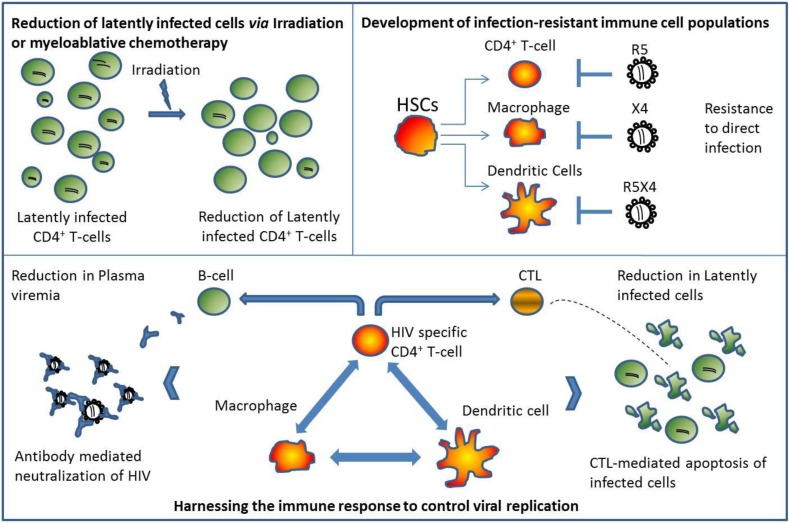
**Autologous Transplantation: Developing an Infection-Resistant Immune System Capable of Controlling Viral Replication. (Top Left Panel)** Decrease in latently infected cells following total body irradiation or myeloablative chemotherapy. Studies have shown that although there is a significant reduction in proviral DNA in peripheral blood in HIV-1 infected patients undergoing autologous transplantation while on continuous Highly-Active-Anti-Retroviral Therapy (HAART), HIV remains detectable in the months following hematopoietic stem cell transplantation (HSCT). **(Top Right Panel) Genetically modified HSCs give rise to infection-resistant immune cells following successful engraftment.** Depending on the method used to render immune cells resistant to infection, genetically modified cells can block HIV-infection regardless of viral tropism. **(Bottom Panel) Protected immune cells provide the necessary means to effectively control viral replication in the absence of HAART.** CTL and B-cell function are enhanced due to CD4+ T-cell helper function resulting in increased CTL-mediated apoptosis of infected cells and improved neutralization of plasma viremia. Overall immune function is restored and maintained following the cessation of HAART.

Numerous studies conducted in animal models and data from human patients have demonstrated the critical importance of CD4+ T-cell help in vivo for the generation of on effective Cytotoxic T-Lymphocyte (CTL)-responses and B-cell functions [66,67]. Despite the high number of HIV-specific CD4+ T-cells in elite controllers, this subset of antigen specific T-cells is maintained and does not serve to fuel viral infection; this is in stark contrast to findings in progressors in which HIV-specific CD4+ T-cells are the primary target following infection [68]. It is therefore likely that developing infection-resistant, HIV-specific CD4+ T-cells may greatly reduce viral pathogenesis possibly leading to the development of a natural controller phenotype (Figure 2). 

In addition to the maintenance of HIV-specific CD4+ T-cells, multiple factors are associated with elite controller status including strong CTL responses due in part to higher TCR avidity, increased pro-activation and expansion cytokines (e.g. IL-2 and IL-21 production), significantly reduced markers of immune exhaustion and highly effective humoral responses [65,68,69,70,71]. Limited examination of cytokine production following SHIV-challenge of pigtailed macaques transplanted with genetically modified HSCs, demonstrated that experimental macaques maintained significantly higher levels of IL-12 compared to control macaques likely playing an important role in the recovery of CD4+ T-cells following the acute phase of disease [21]. In the same study, it was noted that the maintenance of SHIV-specific CD4+ T-cells coincided with broad and enhanced CTL and B-cell function. As suggested previously, intriguing evidence exists supporting the notion that by developing infection-resistant immune cell populations, an artificially created controller phenotype following HSCT may be attainable. The combination of an enhanced immune response with methods to induce viral reactivation (e.g. Vorinostat) may lead to a significant reduction in the viral reservoir, although eliminating the potential for new infections will be critical for the success of such a combination therapy.

Several questions remain with regards to the potential effects of pre-treatment regimens, irradiation and autologous transplantation on viral reservoirs. Latency is established early in viral infection, and hence, developing methods that root-out latent reservoirs will continue to be critical for the development of eradication strategies. It will, however, be necessary to develop therapeutic approaches that are effective regardless of the vast diversity and rapid evolutionary characteristics of HIV both in a broad population base and within an individual HIV-1-infected patient [72]. An abundance of evidence has shown that the ever changing sequence polymorphisms that evolve within an HIV-1-infected patient render CTL, B-cell and NK function suboptimal [72,73,74,75]: Could the maintenance of infection-resistant immune cell populations keep up with the viral evolution?

## 4. Opinion: Reaching for a Cure

Based on the early results, hematopoietic stem cell transplantation as a curative approach for HIV-1-infected patients remains an intriguing possibility. The potential widespread use of this therapy, however, will hinge on the adaptation of this approach in an autologous setting; whether or not a cure can be achieved using one’s on stem cells remains to be determined. The key to addressing this question will be further research in nonhuman primate models, which can be used to assess many of the unknowns about this potentially curative strategy prior to initiating further clinical studies. For instance, it is critical to determine the remaining viral reservoirs following total body irradiation and to determine the threshold of genetically modified cells that are 1) achievable following ex vivo gene modification and 2) needed to control plasma viremia to levels currently obtainable by current antiretroviral therapy. Attributing variations in proviral DNA content and/or CD4+ T-cells counts to the infusion of genetically modified cells will require stringent analysis as autologous transplantation of non-modified HSCs has been shown to significantly impact both variables. Only long-term follow up studies in the absence of HAART will provide the necessary information needed to ascertain the role infection-resistant immune cell populations may have on controlling viral replication; hence, the importance of conducting similar studies in a NHP-based ‘AIDS-patient model’ are imperative. The potential use HSC-transplantation as a curative therapy for HIV-1-infected patients that successfully control plasma viremia with currently available HAART will remain controversial due to the risks involved with this procedure; however, HSC-transplantation remains at the forefront of curative treatment options currently being developed for HIV-1-infected patients. 
